# Molecular and Cellular Mechanisms of Glioblastoma

**DOI:** 10.3390/cells10061456

**Published:** 2021-06-10

**Authors:** Javier S. Castresana, Bárbara Meléndez

**Affiliations:** 1 Department of Biochemistry and Genetics, University of Navarra School of Sciences, 31008 Pamplona, Spain; 2 Molecular Pathology Research Unit, Virgen de la Salud Hospital, 45005 Toledo, Spain; bmelendez@sescam.jccm.es

Glioblastoma is the most malignant primary brain tumor. The therapeutic approach consisting of temozolomide, surgery and radiotherapy has not achieved any sufficient therapeutic improvement in recent years. Hence, it is urgent that we find cell therapies and/or molecular targets that, together with improvements in radiotherapy and chemotherapy, may benefit patients suffering from this devastating tumor. In this Special Issue of *Cells*, entitled “Molecular and Cellular Mechanisms of Cancers: Glioblastoma”, 20 articles on glioblastoma have been published (14 original articles and 6 reviews). Here, we will present a summary of the most relevant results presented in this issue, which undoubtedly have an impact at the level of a better understanding of the cellular and molecular biology of glioblastoma, as well as of possible new therapeutic targets to combat it.

The six reviews touch on interesting topics, such as immunotherapy [1,2], extracellular vesicles [3], energy metabolism [4], translational machinery [5] and the Hippo signaling pathway [6] in glioblastoma.

Immunotherapy is a growing field for the treatment of cancer and, in particular, for glioblastoma patients [1,2]. However, the identification of the patients that can benefit from immunotherapy treatments, as well as the type of immunotherapeutic approach to be used, still need to be improved for successful and effective treatments. Majc et al. [2] point to the possible causes of resistance to immunotherapy: intracranial location, inter- and intra-tumor heterogeneity or the immunosuppressive tumor microenvironment, among others. Adhikaree et al. [1] also point to PD-L1 expression levels, tumor mutations and T-cell immunosuppression as additional causes. The variety of immunotherapeutic strategies that have been applied in glioblastoma treatment are nicely described, together with advanced in vitro (cancer stem cells, organotypic tissue slices, organoids) and animal tumor models [2].

Adhikaree et al. [1] focus their review on the immune checkpoint PD-1/PD-L1 pathway. The use of PD-1/PD-L1 blockade antibodies in combination with chemotherapy and radiotherapy, or as a monotherapy, has been shown to be inefficient in glioblastoma. A better understanding of the barriers to immunotherapy and the mechanisms of immune resistance may help us to design future strategies for immune-related therapies.

Matarredona et al. [3] have reviewed the influence of extracellular vesicles derived from glioblastoma cells on the tumor microenvironment, as well as the possibility that such vesicles may serve as diagnostic markers of glioblastoma, and may also help the stratification of molecular subtypes of this tumor and the study of resistance to chemotherapy and radiotherapy. The extracellular vesicles are not only produced by the tumor, but also by the peritumoral cells that launch the vesicles at the tumor itself, possibly increasing its tumorigenicity.

Van Noorden et al. [4] review the production of energy and reactive oxygen species (ROS) in glioblastoma cells, making a clear difference between how the most differentiated tumor cells and cancer stem cells obtain energy. Differentiated glioblastoma cells mainly use aerobic glycolysis for ATP production without ROS production, whereas glioblastoma stem cells use oxidative phosphorylation for ATP and modest ROS production, due to the hypoxia and quiescence of cancer stem cells. However, in IDH1-mutated glioblastomas, all cells of the tumor use oxidative phosphorylation for ATP and ROS production. Among the possibilities to treat these tumors, we might have a systemic therapeutic inhibition of oxidative phosphorylation, but the anti-cancer effects of ROS production in healthy cells would be inhibited as well. The authors finally suggest removing cancer stem cells out of their hypoxic niches to enable their differentiation and thus increase their sensitivity to radiotherapy and chemotherapy.

Targeting the translational machinery is becoming a new field of research in cancer biology. Digregorio et al. [5] describe cap-dependent and cap-independent translation initiation mechanisms, and indicate some of the targets, e.g., members of the eIF3 family or 4E-BP1, that, if knocked down, could favor the inhibition of the two forms of translation control.

Finally, Masliantsev et al. [6] review the Hippo pathway in glioblastoma. This control pathway prevents the passage to the nucleus of some gene transcription factors that participate in the control of cell proliferation, survival and maintenance of the stem phenotype. Therefore, Hippo, as a whole, behaves as a tumor suppression pathway. Hippo knockdown would make it possible for cells to dedifferentiate to pluripotent cells, and would induce cell death inhibition, therefore creating the tumor-prone subpopulations of cells.

Among the 14 articles, different important issues related to glioblastomas are studied, such as resistance to treatment [7,8,9,10], cancer stem cells [7,8,11], metabolism [8,12], epithelial-to-mesenchymal transition (EMT) [11,13] and ROS [9,12,14].

Cristofaro et al. [7] demonstrated the possibility of inhibiting cell cycle progression and survival in glioma stem cells via the activation of M2 muscarinic receptors, which leads to the inhibition of Notch1 and EGFR expression.

Glioblastoma is a heterogeneous tumor, not only at the cellular and molecular levels, but also at the metabolic level. The possibility of treating this tumor based on phenotypic metabolic differences is increasingly being considered [8], with the hope that by inhibiting metabolic pathways, not only can tumor cells be killed, but they can also be made vulnerable to chemotherapy clinical treatments.

Lo Dico et al. [9] investigated the possible relationship between fluctuations in mitochondrial ROS release to cytoplasm, chaperone-mediated autophagy (CMA) and cytotoxic effects in temozolomide-sensitive and temozolomide-resistant glioblastoma cells.

Su et al. [10] presented the interrelation between an lncRNA (BC200) and an miRNA (miR-218-5p) in glioblastoma, as two molecular agents linked to tumor malignancy and to temozolomide resistance. Thus, the lncRNA is over-expressed, while the miRNA shows a decreased expression in glioblastoma, which means that BC200 would act as an oncogenic factor, while miR-218-5p would behave as a tumor suppressor miRNA. Additionally, BC200 would act as a sponge for miR-218-5p.

Glioblastoma stem cells express high levels of ATP/P2X7 receptors (P2X7R). Ziberi et al. [11] reported that agonists of P2X7R would induce an oncogenic response to cells, with the upregulation of EMT marker expression, and increased cell migration and invasiveness.

Karatsai et al. [12] treated arginine-deprived glioblastoma cells with the arginine analogue canavanine, and detected increased apoptotic cell death only in tumor cells, opening the door to metabolic therapy against glioblastoma.

Hernández-Vega et al. [13] determined that 17β-estradiol, through ER-α, induces EMT by the upregulation of vimentin and N-cadherin expression, and increases migration and invasion in glioblastoma cells.

It is important to have glioblastoma models that allow us to perform reproducible and high-quality studies on glioblastoma biology and treatment. The open access Q-Cell glioblastoma claims to do so. D’Souza et al. [14] made a proteomic analysis of the QCell model and found that the molecular status of the cell lines studied associates with the previously determined transcriptome analysis. This makes it possible to clearly divide cells such as mesenchymal-like or neuronal-like glioblastoma cells, which is undoubtedly important for choosing optimal models of glioblastoma prior to any in vitro research in this tumor.

Vaitkiene et al. [15] made a proteomic analysis of 10 proteins in blood serum from preoperative glioblastoma patients, revealing that the low levels of two of them, osteopontin and IP10, were associated with increased glioblastoma patient survival.

In the present era of biomedical research, experiments using co-cultures and organoid models are very interesting for the evaluation of nanotherapeutics at the experimental level. By combining reconstituted 3D models of glioblastoma (tumoroids) with cerebral organoids and by modulating the activity of microglia with dendritic polyglycerol sulfate (dPGS), Zhang et al. [16] demonstrated that dPGS-treated microglia reduced tumoroid invasiveness. This study proposes the evaluation of well-defined nanostructures in well-characterized human organoids and co-cultures.

Deletions on the 5′-methylthioadenosine phosphorylase (MTAP) gene have been detected in several tumors, although the role of this gene in carcinogenesis is not clear yet. Menezes et al. [17] demonstrated MTAP loss of expression as a frequent genetic event in glioblastoma, but MTAP expression was not associated with survival, which downplays this gene as a potential tumor suppressor gene candidate.

Navarro et al. [18] showed that EGFRvIII mutation and ADD3 loss were bad prognostic markers in a series of glioblastomas studied by multiplex ligation-dependent probe amplification. From this result, we might understand the absence of effect of the inhibitors of receptor tyrosine kinases in those glioblastomas that do not present EGFR mutations.

Alves et al. [19] demonstrated that the WNK2 gene, a known glioma suppressor gene, negatively regulates the migration and invasion of glioblastoma cells and inhibits their autophagic flux.

Lastly, Fellinger et al. [20] presented results on the accumulation of Hsp70 in the membrane of glioblastoma cells after high irradiation doses. Hsp70 can be found overexpressed in the plasma membrane or in the cytosol of tumor cells, including glioblastoma. Depending on its location in the cell, Hsp70 can promote cell growth (cytosolic location) or serve as a target for NK cells (tumor cell membrane location). The localization of this protein in tumor cell membranes and the consequent activation of NK cells suggests the combination of high-dose radiotherapy with immunotherapy treatments. 

In summary, this Special Issue presents 20 articles that help us to better understand the biology of glioblastoma and some possible ways to improve its treatment. Resistance to chemotherapy and radiotherapy, cancer stem cells, energy metabolism, EMT, immunotherapy and ROS are discussed, referring to this deadly tumor.
